# Blanching-Induced Changes in Polyphenol Oxidase, Antioxidants and Phenolic Profile of Mangosteen Pericarp

**DOI:** 10.17113/ftb.62.04.24.8513

**Published:** 2024-12

**Authors:** Giroon Ijod, Nur Izzati Mohamed Nawawi, Rabiha Sulaiman, Noranizan Mohd Adzahan, Farooq Anwar, Ezzat Mohamad Azman

**Affiliations:** 1 Department of Food Technology, Faculty of Food Science and Technology, Jalan Universiti 1, 43400, Universiti Putra Malaysia, Selangor, Malaysia; 2 Department of Food Science, Faculty of Food Science and Technology, Jalan Universiti 1, 43400, Universiti Putra Malaysia, Selangor, Malaysia; 3 Institute of Chemistry, University of Sargodha, University Road, Sargodha-40100, Pakistan

**Keywords:** waste valorisation, stability of anthocyanins, browning enzyme, sustainable processing of cyanidin-3-*O*-glucoside

## Abstract

**Research background:**

Anthocyanin pigments in mangosteen pericarp can serve as natural colourants; however, their stability is compromised by enzymatic browning caused by polyphenol oxidase (PPO). Thus, this study aims to investigate how hot water and steam blanching affect the PPO activity, phenolic profile and antioxidant properties of mangosteen pericarp.

**Experimental approach:**

Fresh mangosteen pericarp was blanched in hot water or steam at 100 °C for 0, 30, 60, 90 and 120 s and the residual PPO activity, total phenolic content (TPC), total anthocyanins, antioxidant activity, browning index and colour properties were evaluated. Additionally, the phenolic compounds were identified using liquid chromatography-mass spectrometry (LC-MS).

**Results and conclusions:**

Zero-order reaction kinetics (R^2^>0.800) showed that residual PPO activity was significantly (p<0.05) reduced in both blanched and steamed mangosteen pericarp. As expected, PPO was inactivated more rapidly in hot water (*t*_1/2_=59.0 s) than in steam blanching (*t*_1/2_=121.1 s). However, the principal component analysis (PCA) showed that steam blanching for 90 s was the most efficient method, preserving the highest levels of antioxidant capacity, expressed as Trolox equivalents (TE; 9135 µmol/g), Fe(III)-reducing power, expressed as TE, (9729 µmol/g), total anthocyanins (3.03 mg/g), and TPC, expressed as gallic acid equivalents (1057 mg/g). Overall, steam blanching for 90 s was the most efficient method because it best preserved the phenolic compounds and is also a cost-effective method compared to hot water, which needs to be replaced after a few applications.

**Novelty and scientific contribution:**

This is the first study to report the effects of blanching on the anthocyanins mainly present in mangosteen pericarp, in particular cyanidin-3-*O*-sophoroside (C3S) and cyanidin-3-*O*-glucoside (C3G), using high-performance liquid chromatography (HPLC) and LC-MS. This study makes a significant scientific contribution to the food industry by providing suitable blanching methods to preserve the quality of bioactive compounds, especially anthocyanins in mangosteen pericarp, which can be used as a natural colourant.

## INTRODUCTION

Mangosteen (*Garcinia mangostana* L.) is a tropical fruit known as the ’queen of fruits‘ (*1*). In 2022, approx. 1.15 million tonnes of mangosteen were produced worldwide, with Thailand, Indonesia, Vietnam and Malaysia making a significant contribution (*2*). Mangosteen flesh is used in various food products, including ice cream, yoghurt, juice, chocolate, lozenges, fruit jam and nutritional supplements (*3*). However, the mangosteen pericarp, which accounts for 60 % of the fruit mass, is typically considered agro-waste (*4*). The consumption and processing of mangosteen generates up to 690 000 metric tonnes of mangosteen pericarp by-products annually, which are often discarded and pollute the environment instead of being used for value-added ingredients (*4*).

Many studies have shown that the mangosteen pericarp is a considerable source of bioactive compounds with a high antioxidant capacity, especially xanthones and anthocyanins (*5*-*7*). For example, α-, β- and γ-mangostin are the main xanthones in mangosteen pericarp, while cyanidin-3-*O*-sophoroside (C3S), cyanidin-3-*O*-glucoside (C3G) and pelargonidin-3-*O*-glucoside (P3G) make up the anthocyanins (*6*, *8*, *9*). Moreover, mangosteen pericarp extracts have antioxidant, anti-inflammatory, anti-cancer and antibacterial properties (*1*, *5*-*7*, *9*-*12*). They have also been shown to reduce the risk of coronary heart disease and atherosclerosis (*7*, *10*-*12*).

Anthocyanins from the mangosteen pericarp can be excellent natural colourants due to their hydrophilic nature and vibrant red-to-purple hues (*13*, *14*). However, the stability of anthocyanins is affected by various factors, including temperature, pH, light, oxygen and enzymatic activity (*15*-*17*). The main problem with mangosteen pericarp is its increased content of an enzyme called polyphenol oxidase (PPO), which causes the pericarp to turn brown quickly. PPO is known as a typical endogenous enzyme due to the presence of a dinuclear copper centre (*18*). PPO accelerates two reactions in the presence of molecular oxygen: the oxidation of *o*-diphenols to form *o*-quinones and the *o*-hydroxylation of monophenols to form *o*-diphenols (*18*). Quinones are highly reactive electrophilic substances that can damage or covalently alter nucleophiles like anthocyanins, which initiate the formation of the brown pigment melanin (*15*). Blanching is necessary as a pretreatment to avoid these problems and to preserve the biological activity and natural colour of anthocyanins (*19*, *20*).

Blanching is a frequently used method to reduce the microbial load and suppress enzymes in fresh fruits, vegetables and grains (*19*, *20*). Traditionally, blanching with hot water or steam is used to prevent PPO activity and thus preserve the majority of enzymatically degradable water-soluble bioactive compounds such as anthocyanins (*20*, *21*). In hot water blanching, materials are typically immersed in hot or boiling water at 80 to 100 °C for 1 to 2 min. In steam blanching, steam at (95±1) °C is applied for 1.5 to 3 min without direct contact with the medium (*20*, *22*).

Some studies have shown that PPO can be suppressed in mangosteen pericarp by using hot water and steam blanching processes. Nevertheless, relatively few studies have focused on the relationship between anthocyanins and PPO activity or the profiling of anthocyanins in extracts. Therefore, the aim of this study is to investigate the effects of PPO inactivation by hot water and steam blanching on the antioxidant properties of mangosteen pericarp, including anthocyanin composition, total phenolics, antioxidant activity, browning index (BI) and colour properties. In addition, a phenolic profile of the extracts from the blanched mangosteen pericarp was established using LC-MS. Pearson correlation, general linear modelling (GLM) and principal component analysis (PCA) were used to evaluate the relationships between the investigated properties.

## MATERIALS AND METHODS

### Chemicals

The following chemicals were obtained from Sigma-Aldrich, Merck (St. Louis, MO, USA): potassium chloride buffer (0.025 M), sodium acetate buffer (0.4 M), 4,6-tripryridyl-*s*-triazine (TPTZ), gallic acid (98 %), Trolox, Folin–Ciocalteu reagent, 2,2-diphenyl-2-picrylhydrazyl (DPPH) and iron(III) chloride hexahydrate. Triton X-100 and polyvinyl pyrrolidone (PVPP) were purchased from Chemiz (Shah Alam, Malaysia). Catechol (99 %) was obtained from Acros Organics, Thermo Fisher Scientific (Newark, NJ, USA). Sodium carbonate and acetic acid were obtained from Systerm (Selangor, Malaysia). Pelargonidin-3-*O*-glucoside (P3G), cyanidin-3-*O*-glucoside (C3G) and cyanidin-3-*O*-sophoroside (C3S) with 99 % purity were purchased from ExtraSynthese Ltd. (Genay, France). Ethanol, formic acid and methanol (HPLC grade) were of analytical grade and bought from Merck (Darmstadt, Germany).

### Sample preparation

The purple mangosteen (index 6, *i.e.* fully ripened fruit) was procured in September 2022 from a nearby market in Serdang, Selangor, Malaysia. The fresh mangosteen was cleaned under running water, and the pericarp was manually separated from the flesh. Mangosteen pericarp was then cut equally into eight pieces before the blanching process.

#### Steam blanching

The steam blanching was conducted using a steamer (SR-Y22FGJ; Panasonic Appliances Co., Ltd., Chennai, India) with 3 L of distilled water preheated to (100±1) °C. Briefly, 20 g of mangosteen pericarp were placed in the steaming basket for 30, 60, 90 and 120 s, respectively. The steamed mangosteen pericarp was immediately transferred to ice water to stop the blanching process.

#### Hot water blanching

The hot water blanching was conducted using a cooker (SR-Y22FGJ; Panasonic Appliances Co., Ltd) with 3 L of distilled water preheated to (100±1) °C. In this process, 20 g of mangosteen pericarp were immersed in hot water for 30, 60, 90 and 120 s, respectively. The blanched pericarp was immediately transferred to the ice water to stop the blanching process.

**Fig. S1:**
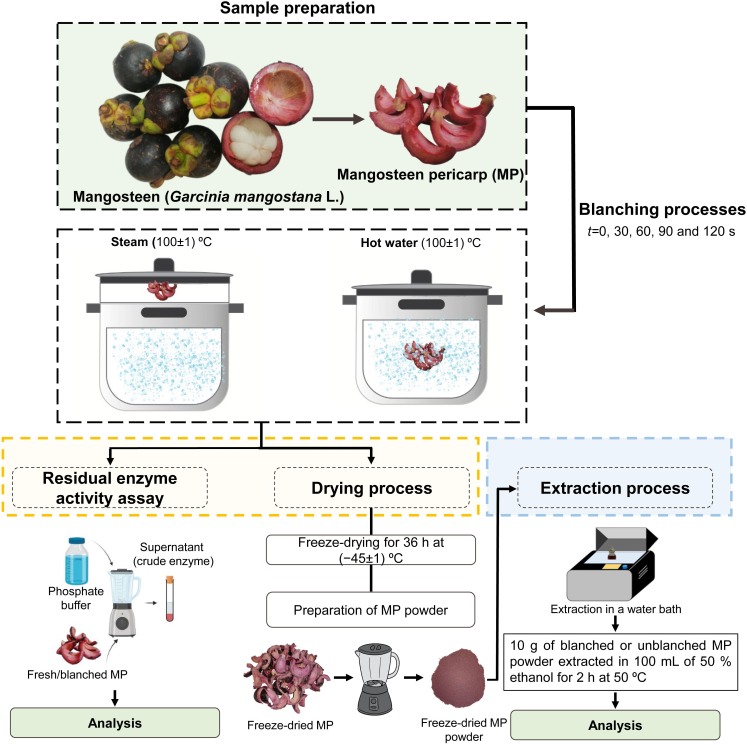
Flow chart of blanching methods and analysis of mangosteen pericarp

For the preparation of dried mangosteen pericarp samples, 600 g of blanched and unblanched mangosteen pericarp were lyophilised in a freeze dryer (Labconco, Kansas City, MO, USA) at (–45±1) °C for 36 h following the method of Nawawi *et al*. (*17*). Dried mangosteen pericarp was ground using a blender (model 32BL79; Waring, McConnellsburg, PA, USA) for 2 min to pass through a 0.50 mm (35 mesh) sieve. All samples were stored at –20 °C until further analysis. The flow chart in Fig. S1 shows the of mangosteen pericarp sample preparation, blanching and analysis.

### Preparation of a crude enzyme extract from the mangosteen pericarp

The crude enzyme extract from the mangosteen pericarp was prepared according to Deylami *et al*. (*15*) with a slight change in the mass ratio of blanched mangosteen pericarp to different buffer volumes. Potassium phosphate buffer (0.1 M, pH=7.5) was prepared and 50 mL of the buffer were used to grind 5 g of mangosteen pericarp (1:10, *V*/*m*) in a blender (32BL79; Waring). In addition, 4 % (*m*/*V*) PVPP and 1 % Triton X-100 were present in the buffer. The mixture was centrifuged for 15 min at 4 °C and 8765×*g* (Sigma 3–18K, Sartorius, Germany). The crude enzyme extract was obtained from the supernatant.

### Residual activity of polyphenol oxidase

The residual activity of polyphenol oxidase (PPO) was determined according to Deylami *et al*. (*15*) with slight adjustments, using different concentrations of substrate and crude enzymes. The substrate solution for PPO activity comprised 1.80 mL of buffer (0.1 M, pH=7.5) and 1 mL of 0.1 M catechol. The reaction was started by adding 0.2 mL of crude enzyme and mixing. The absorbance was measured at 420 nm for 5 min at 1 min intervals using a spectrophotometer (BioMate 3; Thermo Fisher Scientific, Waltham, MA, USA).

The residual enzyme activity (REA/%) was calculated using the following equation:


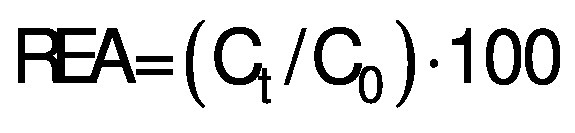
 /1/

where C_t_ and C_0_ were the specific enzyme activities of blanched and unblanched samples, respectively.

### Determination of thermal kinetic parameters

Rate constant (*k*) for zero-order of the kinetic reaction was determined for PPO according to Chisté *et al*. (*23*) using the following equations:


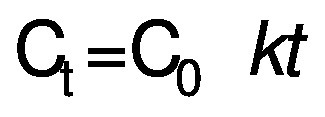
 /2/

where C_0_ is the initial enzyme activity, C_t_ is the corresponding value at the time *t*, *k* is the rate constant and *t* is the heating time (s). The half-life time (*t*_1/2_) for PPO inactivation in both treatments was calculated using the following equation:


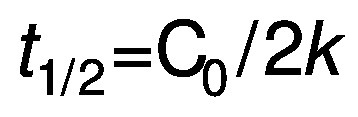
 /3/

where *k* is the zero-order kinetic constant.

### Preparation of dried mangosteen pericarp extract

The dried mangosteen pericarp extracts were prepared following the method previously described by Azman *et al*. (*24*) using a slightly modified solid-to-solvent ratio to increase anthocyanin extraction. The solid-to-solvent ratio was set at 1:10 (*m*/*V*) and dried mangosteen pericarp (10 g) was mixed with 100 mL of 50 % ethanol. The mixture was placed in a water bath (at 50 °C for 2 h and 180 rpm; WITEG Labortechnik, Wertheim, Germany) for extraction. The extract was vacuum-ﬁltered using a Buchner funnel and further filtered using Whatman No. 1 filter paper before centrifugation (model 4200; Kubota, Tokyo, Japan) at 1030×*g* for 15 min. A clear supernatant was collected and stored at −20 °C for further analysis.

### Identification of phenolic compounds by liquid chromatography-mass spectrometry

Liquid chromatography-mass spectrometry (LC-MS) analysis of phenolic compounds was carried out using a Dionex UltiMate 3000 Rapid Separation (RS) ultra-performance liquid chromatography (UPLC) system (Thermo Fisher Scientific Inc.) connected to a Thermo Scientific Q Exactive Orbitrap hybrid tandem mass spectrometer and heated electrospray ionisation II (H-ESI II). Chromatography was carried out using a Purospher STAR RP18 end-capped column (5 µm particle size, 250 mm×4.6 mm i.d.; Merck, Darmstadt) with the column temperature set at 30 °C. A mobile flow rate of 0.8 mL/min and an injection volume of 10 μL were applied. The mobile phase consisted of solvent A (*φ*(formic acid,water)=0.2 %) and solvent B (*φ*(methanol,water)=99.9 %). The following gradient elution methods were used: 15 % B for 0–19 min, 35 % B for 19–38 min, 60 % B for 38–50 min, 80 % B for 50–56 min, and 15 % B for 56 min. Mass spectrometry (MS) spectra were collected between *m*/*z*=100 and 1500 in the positive and negative ion modes, with scan resolutions of 70 000 (full MS scan) and 35 000 (ddMS2 scan). To examine the obtained data for MS analysis, the Qual Browser of Xcalibur^TM^ software v. 4.3 (*25*) was used.

### Determination of anthocyanin content using high-performance liquid chromatography

The HPLC analysis followed Azman *et al*. (*24*) with minor adjustments. The anthocyanin content was determined at 30 °C using a Purospher STAR RP18 end-capped column (250 mm×4.6 mm i.d., particle size 5 µm; Merck, Darmstadt) in a Perkin Elmer Series 200 HPLC system (PerkinElmer Inc., Shelton, CT, USA), equipped with a Perkin Elmer Series 200 UV/Vis detector, Perkin Elmer Series 200 pump, Shimadzu CTO-10A column oven (Shimadzu Corporation, Kyoto, Japan) with a manual injector and Perkin Elmer Series 200 vacuum degasser. Solvent A: formic acid (2 %) and solvent B: methanol (*φ*(methanol,water)=99.9 %) were used as the mobile phase. The gradient elution system was set at 15 % (B) for 0 min, 35 % (B) for 15 min, 60 % (B) for 30 min and 80 % (B) for 40 min. The following parameters were used: flow rate 1.0 mL/min, volume 20 µL, wavelength 520 nm and running time 45 min. The external standard calibration curve was prepared at 0.01–0.1 mg/mL for the quantification of anthocyanins. The determination coefficient (R^2^), the limit of detection (LOD) and limit of quantification (LOQ) were calculated as follows:


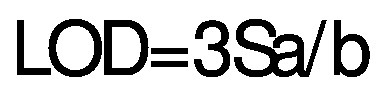
 /4/


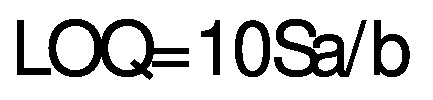
 /5/

where Sa is the standard deviation of the response and b is the slope of the calibration curve.

### Determination of total phenolic content

The Folin-Ciocalteu assay was used to determine the total phenolic content (TPC) following the method by Azman *et al*. (*16*). Gallic acid (0-100 mg/L) was used as a standard. The results were expressed as mg of gallic acid equivalents (GAE) per g. Mean values were calculated from triplicate measurements.

### Determination of antioxidant activity

#### DPPH free radical scavenging activity

The efficacy of dried mangosteen pericarp extracts to scavenge the DPPH free radical was tested using the methods of Ezzat *et al*. (*26*) and Zhou *et al*. (*27*) with some changes, such as the use of different solvents, dilution factors and volume of the extract. DPPH (0.15 mM) was prepared in 250 mL of 80 % ethanol and 0–2000 µM of Trolox standard. Sample/Trolox (0.05 mL) was added in 1.95 mL of 0.15 mM DPPH and vortexed for 10 s. The solution was stored at ambient temperature in the dark for 30 min. A spectrophotometer (BioMate 3; Thermo Fisher Scientific) was used to measure the change in absorption at 517 nm. The results were presented in µmol of Trolox equivalents (TE) per g of dried mangosteen pericarp sample.

#### Fe(III)-reducing antioxidant power assay

Fe(III)-reducing antioxidant power (FRAP) analysis was based on the method of Senevirathna *et al*. (*28*) with some modifications, including diluting the extracts before combining with the FRAP reagent and extending the incubation time to 30 min. The results were calculated using a Trolox solution standard curve (0–2000 µM) and expressed in µmol of TE per g of dried mangosteen pericarp sample.

### Determination of colour properties

A colourimeter (CR-410; Konica, Minolta, Japan) was used to examine the colour of dried mangosteen pericarp extracts. The observed colour parameters were L*, a*, b*, chroma (C*) and hue angle (*h°*). Total colour difference (∆*E*), *h°* and C* were calculated according to the following equations:


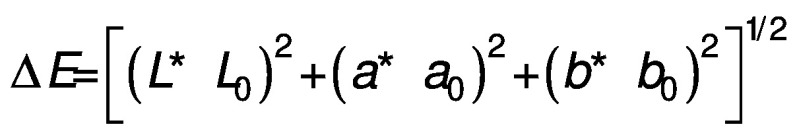
 /6/


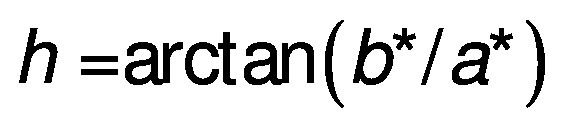
 /7/


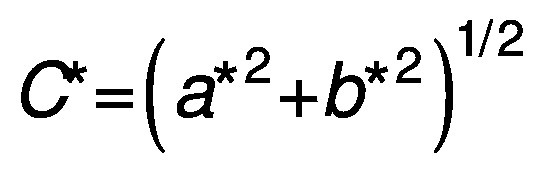
 /8/

where *L*_0_, *a*_0_ and *b*_0_ are blank values of control samples depending on the free anthocyanin concentration.

### Determination of browning index

The browning index (BI) was determined according to Velázquez-Estrada *et al*. (*29*), with slight modifications. Approximately 1.0 g of dried mangosteen pericarp powder was dissolved in 20 mL of 65 % ethanol, held at ambient temperature for 30 min and filtered through Whatman No. 1 filter paper. The absorbance of the extract was measured at a wavelength of 420 nm using a spectrophotometer (BioMate 3; Thermo Fisher Scientific).

### Statistical analysis

All statistical analyses were conducted using one-way and two-way analysis of variance (ANOVA). Tukey’s multiple range tests and *t*-test were used with a significance of p<0.05. The Pearson’s linear correlation was also applied to assess the correlations between the analyses. Statistical and multivariate analyses such as general linear model (GLM) and principal component analysis (PCA) were carried out in Minitab v. 21 (*30*).

## RESULTS AND DISCUSSION

The extraction of bioactive compounds from the dried mass is more reliable than from the fresh mass, as phytochemicals break down immediately under the conditions of high water activity (*31*). Freeze-drying is one of the simplest and most effective methods for drying plant material, especially fruits or agro-waste, with high moisture content. To obtain a powder with a moisture content below 10 % (*m*/*m*), the freeze-drying time for the fresh and blanched mangosteen pericarp was set at 36 h in the present study, based on the results of Nawawi *et al*. (*17*). Consequently, it is recommended that plant-based powders have a moisture content of less than 10 % (*32*) to increase the phytochemical stability of the pigments, prevent microbial growth and reduce the browning processes of enzymatic and non-enzymatic sources (*33*). In addition, GLM analysis revealed significant (p<0.05) effects of blanching, time and their interactions on residual enzyme activity, total anthocyanins, total phenolic content (TPC), antioxidant capacity and colour quality of mangosteen pericarp extracts.

### Residual polyphenol oxidase activity

Inactivation of polyphenol oxidase (PPO) can prevent the discolouration and degradation of bioactive compounds and, thus, the loss of nutritional value of mangosteen pericarp (*15*, *34*). In this study, a significant reduction (p<0.05) of residual PPO activity was detected, following the zero-order reaction kinetics (R^2^>0.800) in both hot water- and steam-blanched mangosteen pericarp. As expected, the inactivation of PPO is faster in hot water (*t*_1/2_=59.0 s) than in steam blanching (*t*_1/2_=121.1 s) (Table 1), which may be related to the immersion of the mangosteen pericarp directly in the heated blanching medium (*35*).

**Table 1:** Residual enzyme activity and kinetic study of polyphenol oxidase (PPO) in hot water and steam blanching

	Residual enzyme activity/%	
*t*(blanching)/s	Hot water	Steam
0 (control)	(100.00±0.00)^Aa^	(100.00±0.00)^Aa^
30	(92.9±0.3)^Ab^	(79.3±1.7)^Bb^
60	(81.0±0.2)^Ac^	(79.0±0.5)^Ab^
90	(18.4±0.3)^Bd^	(61.0±1.3)^Ac^
120	(10.4±0.4)^Be^	(47.2±1.4)^Ad^
Order	Zero	Zero
Rate constant, *k*	0.846	0.413
R^2^	0.880	0.954
*t*_1/2_/s	(59.00±0.01)^B^	(121.1±4.0)^A^

Each result represents the mean value±standard deviation (*N*=3). Values with the same lowercase letter in superscript in the same row are not significantly different (p>0.05). Values with the same capital letter in superscript in the same column are not significantly different (p>0.05)

Deylami *et al*. (*15*) also studied the inactivation of mangosteen pericarp blanched in hot water at 60–100 °C for 12 min. In contrast to this study, the authors discovered that a first-order kinetic model could be fitted to the model. The discrepancies in the results of Deylami *et al*. (*15*) may be due to the different concentrations and types of PPO enzymes in the mangosteen pericarp, which differ in their heat stability. Different concentrations of labile and resistant PPOs affect the inactivation rate. The higher concentration of labile PPO in the study of Deylami *et al*. (*15*) could explain why the first-order model fits, as the labile PPO is more easily inactivated, followed by the resistant PPO. In contrast, our zero-order kinetics suggests the presence of more heat-resistant PPOs that require longer times or higher temperatures for inactivation. Deylami *et al.* (*15*) observed an inactivation of PPO activity of ~77 and ~86 % at 90 and 100 °C, respectively, after 12 min. The results of the current study are in agreement with those of Moscetti *et al*. (*35*), who found that steam blanching potato slices at 90 °C had a longer half-life (0.750 min) than blanching in hot water at 90 °C (0.587 min), suggesting that a longer time is required to inactivate PPO in steam than in hot water.

In the first 60 s of hot water blanching, only ~19.0 % of PPO was inactivated in mangosteen pericarp (Table 1) compared to ~40 % in pitaya peels, as reported by Anh *et al*. (*36*). Different fruits have different cell wall structures; therefore, different blanching times are required to inactivate the PPO (*35*, *37*). In addition, a significantly higher inactivation (p<0.05) of PPO activity was observed after blanching in hot water for 90 and 120 s than after steam blanching. Bernstein and Noreña (*38*) also found that there were no visible changes in the PPO activity of red cabbage after 1 min of steam blanching and significant PPO inactivation (~55.2 %) was achieved only after 10 min at 100 °C, which is a lower inactivation rate than in this work using the same blanching method. However, after 10 min of steam and hot water blanching (80 and 90 °C), PPO activity was significantly reduced to ~48, ~54 and ~55 %, respectively.

A two-sample *t*-test and Tukey’s test were conducted to compare PPO inactivation rates. The two-sample *t*-test revealed no significant differences in PPO inactivation between hot water (mean=60.5 %) and steam (mean=73.3 %) blanching for 2 min (p>0.05). However, the Tukey’s test showed a significant difference, suggesting that multiple comparisons or factors should be considered to better assess the overall variability of the blanching treatments. Overall, steam blanching takes longer than hot water blanching to achieve a similar residual PPO activity, probably because hot water has a higher specific heat capacity than steam (*19*). The presence of different types of isoenzymes, concentrations of enzymes and cell morphological structures may explain the differences in their results and the current research. In addition, different morphologies of red cabbage may require more time to soften the inner core and its cells than mangosteen pericarp cells. Therefore, appropriate blanching is needed, especially when dealing with heat-sensitive compounds such as anthocyanins and other phenolic components in mangosteen pericarp, which should be considered, even if hot water blanching outperforms steam blanching in the inactivation of PPO.

### Liquid chromatography-mass spectrometry analysis

The preliminary study found higher total monomeric anthocyanin content (TMAC) in the mangosteen pericarp blanched in hot water and steam for 90 s (Table S1). The phenolic compounds such as anthocyanins and xanthones were then identified by MS. The identifications were based on the parent ions [M-H]+ and [M-H]- as well as on the fragment ion pattern (MS/MS). As shown in Table 2 (*8*, *9*, *37*, *39*-*44*), the parent ions ranging between *m*/*z*=611.1612 and 611.1617 [M-H]+ and confirmed by fragment ion 287.0529–287.0539 were identified as C3S. The parent ion at *m*/*z*=449.1770–449.1780 [M-H]+ was also tentatively identified as C3G, which was confirmed by the fragment ion at 287.1229–287.1241.

**Table 2:** Anthocyanins and phenolic compounds identified in fresh and in mangosteen pericarp blanched with hot water and steam as detected by LC-MS

No.	*t*_R_/min	Compound name	Formula	*M*	M/z^+^	M/z^-^	MS/MS		Reference
Fresh mangosteen pericarp									
1.	5.39	Quinic acid	C_7_H_12_O_6_	192.1666		191.0408	111.0208, 191.0389, 87.0191, 85.0396	(*37*)	
2.	10.21	β-Mangostin	C_25_H_28_O_6_	424.4930		423.0968	261.0415, 151.0039, 262.0449, 109.0296	(*9*)	
3.	10.82	α-Mangostin	C_24_H_26_O_6_	410.4660		411.0260	241.0021, 96.9593, 169.0132, 411.0226	(*9*)	
4.	14.03	Procyanidin trimer	C_45_H_38_O_18_	866.7724	867.2134		127.0379, 139.0373, 247.0571, 163.0368	(*39*)	
5.	14.68	A-type proanthocyanidin	C_45_H_36_O_18_	864.7650		865.1985	125.0225, 407.0748, 289.0698, 287.0541	(*40*)	
6.	16.56	Procyanidin dimer	C_30_H_26_O_12_	578.5202	579.1576		127.0371, 139.0370, 287.0514, 163.0366	(*39*)	
7.	17.13	Procyanidin B1	C_30_H_26_O_12_	578.5202		577.1406	289.0748, 125.0264, 407.0800	(*41*)	
8.	17.26	Cyanidin-3-*O*-sophoroside	C_27_H_31_O_16_	611.5254	611.1617		287.0539, 288.0571, 287.2075	(*8*, *9*, *39*)	
9.	18.83	Procyanidin trimer	C_45_H_38_O_18_	866.7724	867.2209		127.0383, 247.0578, 139.0381, 245.0423	(*39*)	
10.	19.49	Procyanidin C1	C_45_H_38_O_18_	866.7724		865.1997	125.0231, 289.0709, 407.0758, 287.0550	(*40*)	
11.	21.37	Catechin	C_15_H_14_O_6_	290.2681	291.0936		139.0392, 123.0444, 147.0438, 165.0541	(*37*)	
12.	22.04	(-)-Epicatechin	C_15_H_14_O_6_	290.2681		289.0752	289.0723, 245.0820, 203.0709, 109.0290	(*40*, *42*, *43*)	
13.	27.40	Procyanidin dimer	C_30_H_26_O_12_	578.5202	579.1492		127.0381, 123.0429, 287.0522, 163.0369	(*39*)	
14.	28.08	Procyanidin B1	C_30_H_26_O_12_	578.5202		577.1367	125.024, 289.0718, 407.0770	(*41*)	
15.	29.32	Cyanidin-3-*O*-glucoside	C_21_H_21_O_11_	449.3840	449.1780		287.1236, 449.1766	(*8*, *9*)	
16.	32.56	Dihydroquercetin	C_15_H_12_O_7_	304.2516	305.0647		153.0163, 149.0212, 231.0629, 259.0579	(*44*)	
Mangosteen pericarp blanched in hot water									
1.	5.40	Quinic acid	C_7_H_12_O_6_	192.1666		191.0245	111.0118, 87.0124, 85.0330, 191.0235	(*37*)	
2.	10.22	β-mangostin	C_25_H_28_O_6_	424.4930		423.0952	261.0404, 151.0027, 109.0287, 262.0436	(*9*)	
3.	10.66	α-Mangostin	C_24_H_26_O_6_	410.4660		411.0261	241.0019, 96.9598, 169.0131, 411.0228	(*9*)	
4.	13.96	Procyanidin trimer	C_45_H_38_O_18_	866.7724	867.2133		127.0400, 139.0400, 163.0399, 247.0596	(*39*)	
5.	14.60	A-type Proanthocyanidin	C_45_H_36_O_18_	864.7650		865.1974	125.0230, 407.0751, 289.0702, 287.0550	(*40*)	
6.	16.32	Procyanidin dimer	C_30_H_26_O_12_	578.5202	579.1642		127.0403, 139.0401, 287.0551, 163.0399	(*39*)	
7.	17.11	Procyanidin B1	C_27_H_31_O_16_	578.5202		577.1394	287.0529, 288.0566	(*41*)	
8.	17.14	Cyanidin-3-*O*-sophoroside	C_30_H_26_O_12_	611.5254	611.1606		289.0750, 125.0268, 407.0797, 245.0841	(*8*, *9*, *39*)	
9.	18.59	Procyanidin trimer	C_45_H_38_O_18_	866.7724	867.2139		127.0389, 247.0584, 139.0385, 163.0379	(*39*)	
10.	19.50	Procyanidin C1	C_45_H_38_O_18_	866.7724		865.1999	125.0269, 289.0744, 407.0787, 287.0585	(*40*)	
11.	20.87	Catechin	C_15_H_14_O_6_	290.2681	291.0931		139.0394, 123.0445, 147.0445, 165.0544	(*37*)	
12.	21.96	(-)-Epicatechin	C_15_H_14_O_6_	290.2681		289.0796	289.0724, 245.0821, 203.0713, 109.0289	(*40*, *42*, *43*)	
13.	27.01	Procyanidin dimer	C_30_H_26_O_12_	578.5202	579.1505		127.0390, 123.0442, 287.0542, 139.0391	(*39*)	
14.	27.83	Procyanidin B1	C_30_H_26_O_12_	578.5202		577.1354	125.0234, 289.0708, 407.0766, 161.0235	(*41*)	
15.	29.28	Cyanidin-3-*O*-glucoside	C_21_H_21_O_11_	449.3840	449.1788		287.1241, 449.1778	(*8*, *9*)	
16.	32.25	Dihydroquercetin	C_15_H_12_O_7_	304.2516	305.0660		153.0175, 231.0642, 149.0227, 259.0586	(*44*)	
Mangosteen pericarp blanched with steam									
1.	5.31	Quinic acid	C_7_H_12_O_6_	192.1666		191.0246	111.0121, 87.0125, 85.0332, 191.0236	(*37*)	
2.	10.20	β-Mangostin	C_25_H_28_O_6_	424.4930		423.1003	261.0456, 151.0074, 109.0332, 262.0486	(*9*)	
3.	10.82	α-Mangostin	C_24_H_26_O_6_	410.4660		411.0257	241.0024, 96.9596, 169.0137, 242.0047	(*9*)	
4.	13.98	Procyanidin trimer	C_45_H_38_O_18_	866.7724	867.2129		127.0376, 163.0367 139.0373, 247.0573, 271.0573	(*39*)	
5.	14.58	A-type proanthocyanidin	C_45_H_36_O_18_	864.7650		865.1986	125.0230, 407.0749, 289.0706, 287.0548	(*40*)	
6.	16.25	Procyanidin dimer	C_30_H_26_O_12_	578.5202	579.1537		127.0398, 139.0396, 287.0547, 163.0391	(*39*)	
7.	17.04	Procyanidin B1	C_30_H_26_O_12_	578.5202		577.1409	289.0761, 125.0278, 407.0806, 245.0850	(*41*)	
8.	17.13	Cyanidin-3-*O*-sophoroside	C_27_H_31_O_16_	611.5254	611.1612		287.0535, 288.0568	(*8*, *9*, *39*)	
9.	18.79	Procyanidin trimer	C_45_H_38_O_18_	866.7724	867.2134		127.0384, 247.0577, 139.0382, 163.0376	(*39*)	
10.	19.49	Procyanidin C1	C_45_H_38_O_18_	866.7724		865.2009	125.0275, 289.0751, 407.0798, 287.0596	(*40*)	
11.	21.32	Catechin	C_15_H_14_O_6_	290.2681	291.0923		139.0393, 123.0445, 147.0442, 165.0543	(*37*)	
12.	21.86	(-)-Epicatechin	C_15_H_14_O_6_	290.2681		289.0756	289.0718, 245.0818, 109.0288, 203.0709	(*40*, *42*, *43*)	
13.	27.27	Procyanidin dimer	C_30_H_26_O_12_	578.5202	579.1522		127.0445, 123.0396, 287.0540, 139.0392	(*39*)	
14.	27.90	Procyanidin B1	C_30_H_26_O_12_	578.5202		577.1367	125.0229, 289.0708, 407.0757, 245.0802	(*41*)	
15.	29.45	Cyanidin-3-*O*-glucoside	C_21_H_21_O_11_	449.3840	449.1770		287.1229, 449.1767	(*8*, *9*)	
16.	32.51	Dihydroquercetin	C_15_H_12_O_7_	304.2516	305.0666		153.0182, 231.0645, 149.0230, 259.0595	(*44*)	

*M*=molecular mass, *t*_R_=retention time, M/z+=parent ion in positive mode, M/z-=parent ion in negative mode, MS/MS=fragment ion

However, in contrast to Zarena and Sankar (*8*), who found 6.2 % P3G in mangosteen pericarp, P3G was not detected in this study. The variations may be attributed to the different solvents used. For example, Zarena and Sankar (*8*) extracted the mangosteen pericarp with acidified ethanol (0.01 % HCl), which increases the release of P3G. In this study, only a 50 % aqueous ethanolic solution was used. P3G was similarly detected by Li *et al*. (*7*) under optimal conditions (*m*(solid):*V*(solvent)=1:50 mg/mL, pH=2, 80 °C, 2 h). Moreover, the delphinidin-*O*-pentoside identified by Albuquerque *et al*. (*39*) was also not detected in this study, most likely because they extracted the mangosteen pericarp with acidified ethanol (0.1 %, 1 µM citric acid). This indicates that the content and composition of phenolic compounds can be significantly influenced by the methods used for extraction and purification. In addition to anthocyanins, xanthones, phenolic acids and flavonoids are also important phenolic compounds in fresh, hot water- and steam-blanched mangosteen pericarp.

Both blanching methods significantly affected the relative composition of the polyphenols in fresh and blanched mangosteen pericarp (Table S2). Some anthocyanins and catechins were drastically degraded after hot water blanching. Anthocyanins and catechins are heat-sensitive polyphenolic compounds with high solubility in water. Under conditions such as immersion in hot water, these compounds diffuse from the cell matrix into the blanching medium and trigger the opening of their stable flavylium cation of anthocyanins, disrupting the C_6_-C_3_-C_6_ structures. In the case of catechins, hydrolysis and oxidation possibly occur, triggering changes in the flavan-3-ol skeletons and leading to degradation (*13*, *45*). Meanwhile, higher amount of catechin, procyanidin trimer, procyanidin B1, C3S and C3G during steam blanching is due to the heat transfer through vapour, which minimises contact with water and thus preserves most compounds. The trends for anthocyanins were consistent for both types of ionisations. Typically, C3S has a higher molecular mass than C3G and is drastically degraded, which may be due to reduced glucose units (*44*).

The degradation of dihydroquercetin, quinic acid, β-mangostin, α-mangostin, A-type proanthocyanidins and procyanidin C1 was observed in both blanching treatments. However, the extent of degradation was more pronounced in hot water blanching than in steam blanching, with the exception of dihydroquercetin. The breakdown of heat-sensitive phenolics in mangosteen pericarp is believed to occur because of oxidation and the Maillard reaction, which leads to a loss of bioactive substances. The procyanidin trimer and procyanidin B1 were improved in both blanching treatments, while an increase in (-)-epicatechin and procyanidin dimer was only observed after hot water treatment and was significantly reduced after steam treatment. The large amount of (-)-epicatechin and procyanidin dimer might be bound to the cells, and hot water was involved in changing the cell structure by breaking the bonds in the cells, supporting the release of the conjugated compounds during the treatment by enzymatic hydrolysis (*42*, *46*). The reduction of the latter compounds in steam can be associated with degradation during blanching. The latent heat of steam accelerated this process. However, the extractability of the bound (-)-epicatechin and the procyanidin dimer might be slower than in hot water treatment, which would explain the better preservation of these compounds in hot water.

### Anthocyanins

**Fig. 1:**
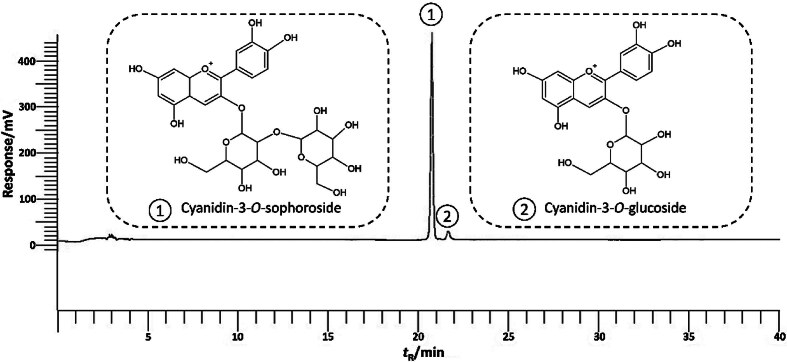
Typical anthocyanin peaks in the extract of mangosteen pericarp measured using HPLC at *λ*=520 nm

The HPLC results showed significantly (p<0.05) more C3S (98.0 %) than C3G (2.0 %) (Fig. 1 and Table 3). This is consistent with the study of Li *et al*. (*7*), who found that C3S (76.1 %) and C3G (13.4 %) were the two most abundant anthocyanins in the mangosteen pericarp. Nevertheless, C3G was below the detection limit after 30 and 120 s of hot water blanching. This suggests that the anthocyanins of mangosteen pericarp are better preserved by steam blanching. Moreover, non-acylated anthocyanins, such as C3G and P3G, are susceptible to degradation due to their heat sensitivity (*13*, *17*). Meanwhile, Deylami *et al.* (*15*) studied the effects of blanching mangosteen pericarp in hot water and reported that the concentration of TMAC increased significantly after 12 min. Nevertheless, there is only a limited study documenting how blanching affects certain anthocyanins in mangosteen pericarp, particularly C3S and C3G.

**Table 3:** Anthocyanin composition of mangosteen pericarp after hot water and steam blanching as measured using HPLC

	*w*(anthocyanin)/(mg/g)			
Treatment	C3S	C3G	P3G	Total
Control	(2.05±0.00)^Ad^	(0.04±0.02)^Bab^	nd	(2.09±0.02)^d^
HW30	(2.44±0.00)^Abcd^	<LOD	nd	(2.44±0.00)^bcd^
HW60	(2.8±0.2)^Aab^	(0.08±0.01)^Ba^	nd	(2.9±0.3)^ab^
HW90	(2.77±0.01)^Aabc^	(0.06±0.02)^Bab^	nd	(2.82±0.01)^abc^
HW120	(2.83±0.02)^Aab^	<LOD	nd	(2.84±0.02)^abc^
ST30	(2.54±0.00)^Aabc^	(0.08±0.01)^Ba^	nd	(2.61±0.01)^abc^
ST60	(2.3±0.1)^Acd^	(0.05±0.03)^Bab^	nd	(2.4±0.1)^cd^
ST90	(2.94±0.06)^Aa^	(0.09±0.00)^Ba^	nd	(3.03±0.07)^a^
ST120	(2.8±0.2)^Aab^	(0.08±0.01)^Ba^	nd	(2.9±0.2)^ab^
LOD/(mg/mL)	0.01	0.01	0.01	
LOQ/(mg/mL)	0.03	0.02	0.02	

Each result represents the mean value±standard deviation (*N*=2). Values with the same lowercase letter in superscript in each column or row are not significantly different (p>0.05). According to Tukey's test, values with the same capital letter in superscript in each row are not significantly different (p>0.05). HW30, 60, 90 and 120=hot water blanching during *t*=30, 60, 90 and 120 s, ST30, 60, 90 and 120=steam blanching during *t*=30, 60, 90 and 120 s, C3S=cyanidin-3-*O*-sophoroside, C3G=cyanidin-3-*O*-glucoside, P3G=pelargonidin-3-*O*-glucoside, nd=not detected, LOD=limit of detection, LOQ=limit of quantification, control=no blanching

The lowest anthocyanin concentration in the control group was probably due to the increased PPO content, which facilitated the browning and degradation of anthocyanins in the mangosteen pericarp (Table 3). According to Deylami *et al*. (*15*), PPO is the most common browning and degrading enzyme in plants, and tropical fruits like mangosteen have high PPO activity. The anthocyanins in the mangosteen pericarp were rapidly degraded due to the production of *o*-quinone by PPO through its interactions with phenolic acids before it was combined with anthocyanins (*13*, *43*).

Overall, the mangosteen pericarp that was blanched with steam (90 and 120 s) and hot water (60, 90 and 120 s) had a higher anthocyanin content than the unblanched mangosteen pericarp (p<0.05). Interestingly, despite the aforementioned interaction of blanching and time to overcome anthocyanins in unblanched mangosteen pericarp, there were no statistically significant differences (p>0.05) among the applied blanching treatments and duration, suggesting that both blanching methods can preserve anthocyanins from degradation at some point. However, other properties (enzyme activity, phenolic content, antioxidants and colour) also need to be considered when valorising the anthocyanins of mangosteen pericarp as a value-added product. Higher anthocyanin concentrations in steam (90 and 120 s) and hot water (60, 90 and 120 s) can be explained by the degradation of pectin, cellulose and hemicellulose and loosening of the cell wall pores. This allows the heat to be rapidly transferred through the cell wall into the cell, which leads to the extraction of large amounts of anthocyanins from the mangosteen pericarp matrix cell (*13*, *47*). Consequently, the anthocyanins in the vacuoles became more accessible.

Instead of hot water blanching, steam blanching can reduce the chance of nutrients leaking into the water, although it has limited penetration in the mangosteen pericarp cell wall. The direct contact of the mangosteen pericarp with the hot water during hot water blanching may have reduced the total concentration of anthocyanins compared to steaming, as they are water-soluble. PPO and total anthocyanins showed a significant negative correlation (r=–0.608, p<0.05), indicating that increased PPO activity led to the degradation of anthocyanins in mangosteen pericarp.

### Total phenolic content

The blanching time and temperature had an effect on the TPC. The TPC increased significantly (p<0.05) at 90 s, but started to decrease at 120 s during both hot water and steam blanching (Table 4). These results are consistent with those of Kim *et al*. (*48*), who observed a comparable trend of TPC after blanching of samnamul in hot water (98 °C, 30 s), resulting in a remarkable increase in TPC, expressed as GAE (93.75 g/kg). Most heat-sensitive phenolic compounds can decompose or leach into the hot water during blanching, resulting in a loss of phenolic compounds (*47*).

**Table 4:** Total phenolic content (TPC), antioxidant capacity and reducing power of mangosteen pericarp in hot water and steam blanching

Treatment	TPC as *w*(GAE)/(mg/g)	Antioxidant capacity as *b*(TE)/(µmol/g)	Reducing power as *b*(TE)/(µmol/g)
Control	(264±15)^g^	(7902±37)^f^	(6439±254)^e^
HW30	(345.7±9.5)^f^	(8088±17)^e^	(8709±71)^cd^
HW60	(557±15)^e^	(8166±20)^e^	(8824±21)^c^
HW90	(937±29)^b^	(8890±42)^b^	(9554±64)^ab^
HW120	(669.4±9.5)^d^	(8666±37)^c^	(9059±99)^bc^
ST30	(390±13)^f^	(8488±34)^d^	(8294±127)^d^
ST60	(595.2±9.5)^de^	(8682±20)^c^	(8724±134)^cd^
ST90	(1057±19)^a^	(9136±23)^a^	(9729±14)^a^
ST120	(800±23)^c^	(8938±42)^b^	(8749±85)^cd^

Each result represents the mean value±standard deviation (*N*=3). Values with the same lowercase letter in superscript in each row are not significantly different (p>0.05). HW30, 60, 90 and 120=hot water blanching during *t*=30, 60, 90 and 120 s, ST30, 60, 90 and 120=steam blanching during *t*=30, 60, 90 and 120 s, GAE=gallic acid equivalents, TE=Trolox equivalents, control=no blanching

Regarding blanching time, both blanching methods resulted in a higher TPC after 90 s. However, steam blanching at 90 s resulted in a significantly higher TPC of ~1.13-fold in mangosteen pericarp than hot water blanching (p<0.05). The cell wall of mangosteen pericarp is mainly composed of lignin, cellulose and hemicellulose. At this point, it can be assumed that both blanching methods altered the membrane and cell wall composition of mangosteen pericarp by depolymerisation of lignin. This phenomenon disrupted the linked bonds in the cell wall, making the membrane porous and promoting the mobilisation of conjugated phenolic substances in the mangosteen pericarp (*46*). In addition, the change in the cell wall structure also facilitated heat transfer in the mangosteen pericarp, resulting in a dual effect of inactivation of PPO and improvement of the extraction efficiency of phenolic compounds.

Nevertheless, a decrease in TPC was observed after 120 s of hot water (~28.6 %) and steam blanching (~24.3 %), compared to 90 s. These findings suggest that certain heat-sensitive and water-soluble compounds responsible for antioxidant activity were degraded after 120 s of blanching. Kim *et al*. (*48*) pointed out that high temperatures and long blanching time could reduce nutrients and antioxidant activities after reaching the threshold. The reduction of TPC in hot water is probably due to the loss of polar-type phenols to the blanching medium (*47*).

Unblanched mangosteen pericarp had lower TPC, expressed as GAE (264 mg/g), which could be due to the high PPO content in the cells that interacted with the phenolic molecules during the extraction. The formation of other compounds could result from this interaction, and the amount of phenolic substances that can react with the Folin-Ciocalteu reagent could be limited (*35*). A strong positive correlation (p<0.05) between the TPC and total anthocyanins (r=0.760), and a negative correlation between the TPC and PPO activity (r=–0.687) indicate that anthocyanins contribute to the TPC and the inactivation of PPO preserves the phenolic compounds in the mangosteen pericarp.

### Antioxidant activity

Mangosteen pericarp is rich in bioactive compounds known for their strong antioxidant properties, including anthocyanins and xanthones (*7*, *49*). Compared to unblanched mangosteen pericarp, steam blanching resulted in significantly (p<0.05) higher antioxidant capacity (~1.5-fold) than hot water (~1.4-fold) after 90 s (Table 4). The trend in antioxidant capacity followed a similar pattern as observed in the TPC results. This could be attributed to the loss of polar-type antioxidants when in contact with hot water during blanching. In contrast, more antioxidants were retained in the steam-blanched samples (*47*). Bernstein and Noreña (*38*) found that red cabbage lost 34.2, 25.1 and 8.8 % of its antioxidant activity when blanched with hot water (80 and 90 °C) and steam (100 °C), respectively. Contrary to the results of Noreña and Rigon (*45*), blanching with steam and hot water at 80 and 90 °C for 10 min resulted in a reduction of antioxidant activity of approx. 74.4, 38.96 and 47.94 %, respectively. Therefore, it is important to avoid excessive pretreatment during blanching to reduce the loss of nutrients and phenolic compounds.

The reducing power of mangosteen pericarp was evaluated using the FRAP assay, which measures the ability to convert the light blue colour of the Fe(III) complex to a dark blue colour of Fe(II) complex (*50*). Table 4 shows that the reducing power increased from 0 to 90 s during both blanching methods and then decreased at 120 s. This indicates that a blanching time of 90 s is optimal to preserve the antioxidant compounds in mangosteen pericarp before they are degraded or leached out. Haw *et al*. (*22*) found that the combination of steaming for 10 min followed by oven drying at 120 °C for 1 h resulted in a 1.21-fold improvement in reducing power compared to the control sample. Kim *et al*. (*48*) also showed that the activity of reducing power increased when samnamul was blanched in hot water at 80, 90 and 98 °C for 30, 45 and 60 s.

Blanching at high temperatures and for a longer time can degrade antioxidants rather than preserve them. Remarkably, the increase in phenolic compounds and reducing sugar, which supports antioxidant activity, is also probably responsible for the increase in antioxidant capacity in the blanched samples (*21*). Reducing power and antioxidant capacity were shown to be strongly correlated (p<0.05) with total anthocyanins (r=0.792, r=0.677) and TPC (r=0.791, r=0.906). These results suggest that anthocyanins and TPC are responsible for the high antioxidant activity and reducing power in mangosteen pericarp. The antioxidant potential of mangosteen pericarp can be attributed to its abundant bioactive compounds, including xanthones, anthocyanins and phenolic acids (*7*, *39*).

### Colour characteristics

L*, a* and b* indicate the lightness, redness and yellowness of the mangosteen pericarp extracts, respectively. As shown in Table 5, the highest a* values were observed during steam blanching at 90 s, indicating a higher redness. A significantly strong positive correlation (p<0.05) between total anthocyanins and a* values (r=0.632) during steam blanching indicates that anthocyanins increased the a* values of mangosteen pericarp at 90 s. At the same time, the highest b* values were observed in hot water at 30 and 60 s (p<0.05), indicating increased yellowness of the mangosteen pericarp extracts.

**Table 5:** Colour characteristics (L*, a*, b*), chroma (C*), hue (*h°*), total colour difference (Δ*E*) and browning index (BI) of extracts from mangosteen pericarp after steam or hot water blanching at different times

Treatment	L*	a*	b*	C*	*h°*	Δ*E*	BI (*A*_420 nm_)
Control	(27.3±0.2)^e^	(21.6±0.3)^d^	(7.8±0.2)^de^	(23.0±0.3)^c^	(19.8±0.2)^c^	-	(1.79±0.00)^a^
HW30	(30.9±0.4)^b^	(30.7±0.5)^b^	(13.0±0.3)^a^	(33.3±0.6)^a^	(22.9±0.1)^b^	(61.4±8.7)^a^	(1.78±0.02)^ab^
HW60	(32.2±0.6)^a^	(30.1±0.8)^b^	(12.8±0.5)^a^	(32.7±0.9)^a^	(23.0±0.3)^b^	(10.5±1.2)^c^	(1.74±0.02)^abcd^
HW90	(27.8±0.3)^de^	(24.0±0.5)^c^	(11.5±0.3)^b^	(26.6±0.5)^b^	(25.6±0.5)^a^	(6.6±2.3)^c^	(1.67±0.02)^cd^
HW120	(29.4±0.1)^c^	(24.0±0.4)^c^	(7.2±0.1)^de^	(25.1±0.3)^b^	(16.8±0.3)^e^	(39.9±4.5)^b^	(1.66±0.00)^d^
ST30	(27.3±0.3)^e^	(20.8±0.3)^d^	(6.9±0.3)^e^	(21.9±0.4)^c^	(18.4±0.4)^d^	(0.8±0.5)^c^	(1.75±0.00)^abc^
ST60	(28.6±0.2)^cd^	(24.2±0.5)^c^	(9.8±0.4)^c^	(26.1±0.6)^b^	(22.1±0.5)^b^	(10.9±1.1)^c^	(1.75±0.06)^abcd^
ST90	(30.4±0.2)^b^	(32.8±0.1)^a^	(9.7±0.3)^c^	(34.2±0.1)^a^	(16.4±0.5)^e^	(69.38±4.2)^a^	(1.65±0.03)^cd^
ST120	(28.4±0.4)^c^	(23.8±1.0)^c^	(8.2±0.6)^d^	(25.2±1.1)^b^	(18.9±0.5)^cd^	(4.1±3.3)^c^	(1.65±0.07)^bcd^

Each result represents the mean value±standard deviation (*N*=3). Values with the same lowercase letter in each row are not significantly different (p>0.05). HW30, 60, 90 and 120=hot water blanching during *t*=30, 60, 90 and 120 s, ST30, 60, 90 and 120=steam blanching during *t*=30, 60, 90 and 120 s, control=no blanching

The lowest L* values in the control and samples steam-blanched for 30 s also contributed to significantly low b* and chroma values (p<0.05). Also, the lowest a* values in control and steam at 30 s were due to high residual PPO activity in the unblanched and shortly blanched mangosteen pericarp, which contributed to high BI of the mangosteen pericarp extract. According to Azman *et al*. (*24*), hue is used to distinguish between red, blue, yellow and green, while chroma indicates the intensity of a colour. At 90 s, the steamed mangosteen pericarp had a significantly higher chroma value, which was consistent with high a* values. However, in addition to the anthocyanin content, Azman *et al*. (*16*) reported that the colour properties and the presence of other compounds during the extraction of the mangosteen pericarp probably affect the high colour intensity.

Furthermore, the highest hue value in hot water was observed at 90 s (~25.57°), which is comparable to the FD&C Red 40 (25°) reported by Wrolstad (*51*). At 90 s, the steam had higher Δ*E* (p<0.05) than the control. No significant difference (p>0.05) was found between Δ*E* and total anthocyanins. This suggests that the colour of the anthocyanins was not the primary factor contributing to the increased Δ*E*, but rather the lowest residual PPO activity in hot water at 120 s.

### Browning Index

According to Karam *et al*. (*31*), BI is crucial for enzymatic and non-enzymatic browning activity. Table 5 shows that the BI values decreased with increasing duration of blanching in hot water or steam. The data showed that unblanched mangosteen pericarp had the highest BI, while a decrease of approx. 2.79, 6.70 and 7.26 % was observed 60, 90 and 120 s after hot water blanching (p<0.05).

In agricultural products, browning is often associated with the enzymatic or non-enzymatic Maillard reaction (*15*, *18*, *31*). The mangosteen pericarp that was blanched with steam and hot water had a lower BI than the unblanched samples. This is probably due to a conformational structural disruption during blanching, which leads to the breakdown of PPO enzyme. According to Deylami *et al*. (*15*), a higher BI in unblanched mangosteen pericarp is caused by an increased PPO activity on the phenolic compounds, which accelerates the browning and results in the intensive production of the brownish colour. Furthermore, the Maillard reaction between the reducing sugar compounds and amino acids in mangosteen pericarp is most likely the cause of brown colour formation (*18*, *31*).

The intense red colour of the mangosteen pericarp extracts was attributed to the preservation of anthocyanins by the inhibition of PPO enzyme, as shown by the significant negative correlation (p<0.05) between total anthocyanins in mangosteen pericarp during hot water and steam blanching (r=–0.612). The reduction of BI in mangosteen pericarp extract was found to correlate with high anthocyanin concentration, TPC and antioxidant activity. Strong negative correlations (p<0.05) were also observed between BI and total anthocyanins (r=–0.608), TPC (r=–0.783), antioxidant capacity (r=–0.801) and reducing power (r=–0.626).

### Principal component analysis

**Fig. 2:**
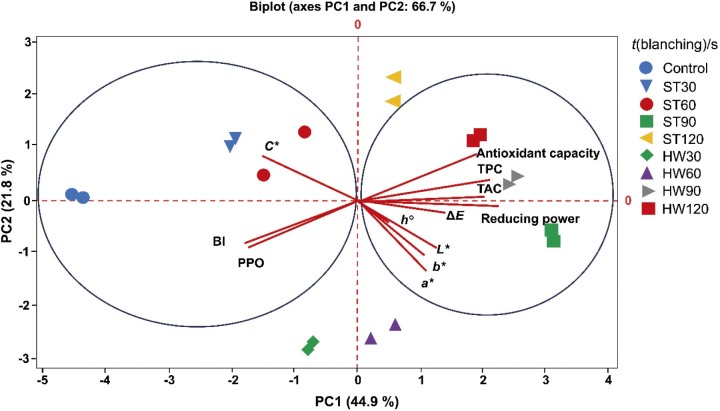
Biplot of principal component analysis based on physicochemical properties, phenolic content, browning index and antioxidant activities of mangosteen pericarp extracts during different blanching methods and blanching times. BI=browning index, TPC=total phenolic content, TAC=total anthocyanin content, PPO=polyphenol oxidase, Δ*E*=total colour difference, L*=lightness/darkness, a*=redness/greenness, b*=yellowness/blueness, C*=chroma, *h°=*hue, ST=steam blanching, HW=hot water blanching

The PCA was used to examine the effects of blanching time and method on total anthocyanins, TPC, antioxidant capacity and colour properties (L*, a*, b*, *h°*, C* and Δ*E*) of mangosteen pericarp. The variables and observations were assigned to the first two principal components, which accounted for 66.7 % of the total variation. As shown in Fig. 2, the first (PC1) and second (PC2) dimensions explained 44.9 and 21.8 % of the total correlation for the mangosteen pericarp samples, respectively. The positive scores on PC1 corresponded to the mangosteen pericarp blanched in hot water at 60, 90 and 120 s and with steam at 90 and 120 s. These variables were associated with high L*, a*, b*, Δ*E*, *h°*, TPC, total anthocyanins, reducing power and antioxidant activity. In contrast, the negative scores on PC1 corresponded to high PPO, BI and chroma values observed in the control, hot water at 30 s and steam blanching at 30 and 60 s. Overall, the results suggest that both blanching treatments for 90 and 120 s, with particular emphasis on steaming for 90 s, appear to be the most effective methods for preserving the colouring phenolic compounds and antioxidant properties of mangosteen pericarp extracts.

## CONCLUSIONS

The blanching method, blanching time and their interactions significantly affected the antioxidant properties, phenolic compound profile and colour intensity of mangosteen pericarp extracts. In this study, hot water blanching appeared to be a better pretreatment for inactivation of polyphenol oxidase (PPO) activity than steam blanching. However, direct contact with hot water significantly damaged the heat-sensitive substances in mangosteen pericarp, especially anthocyanins. Therefore, steam blanching for 90 s was the most efficient process, retaining the highest total anthocyanins, total phenolic content, antioxidant activity and colour intensity. Steam blanching not only helps preserve phenolic compounds but is also a cost-effective method compared to hot water, which needs to be replaced after a few applications. The drastic increase of procyanidin trimer and procyanidin B1 in both blanching treatments was probably the main factor for the antioxidant capacity of mangosteen pericarp. In general, these results could be useful for the food, cosmetic and nutraceutical industries as they can use the anthocyanins of mangosteen pericarp, particularly C3S, as natural colourants.

Since the inactivation of PPO with hot steam takes longer than with hot water, future research should consider times longer than 120 s. In addition, future research should focus more on developing innovative methods to effectively inactivate PPO while maximising the preservation of heat-sensitive components, thereby improving the overall quality of mangosteen pericarp. Investigating the roles of cyanidin-3-*O*-sophoroside and cyanidin-3-*O*-glucoside in mangosteen pericarp from a nutra-pharmaceutical perspective is also crucial.

## Appendix

**Table S1:** Preliminary study on total monomeric anthocyanin content (TMAC) of mangosteen pericarp extracts

	Control	HW30	HW60	HW90	HW120	ST30	ST60	ST90	ST120
*w*(TMAC)/(mg/g)	(1.2±0.1)^e^	(1.56±0.05)^cd^	(1.36±0.06)^de^	(1.69±0.08)^bc^	(1.5±0.1)^cd^	(1.6±0.1)^bc^	(1.71±0.04)^bc^	(2.40±0.09)^a^	(1.88±0.05)^b^

Each result is the mean value±standard deviation (*N*=3). Values with the same lowercase letter are not significantly different (p>0.05). HW30, 60, 90 and 120=hot water blanching during *t*=30, 60, 90 and 120 s, ST30, 60, 90 and 120=steam blanching during *t*=30, 60, 90 and 120 s, Control=no blanching

**Table S2:** Composition of compounds in the fresh, hot water- and steam-blanched extracts of mangosteen pericarp detected by LC-MS

No.	*t*_R_/min	Compound name	*w*(compound)/(g/100 g)		
Fresh	Hot water	Steam
Positive mode					
1.	13.99	Procyanidin trimer	4.68	7.74	10.09
2.	16.56	Procyanidin dimer	20.78	28.25	14.55
3.	17.26	Cyanidin-3-*O*-sophoroside	10.34	3.77	14.64
4.	18.83	Procyanidin trimer	11.01	21.03	20.28
5.	21.37	Catechin	14.60	12.22	17.34
6.	27.40	Procyanidin dimer	15.36	9.75	11.06
7.	29.32	Cyanidin-3-*O*-glucoside	0.12	0.11	1.45
8.	32.56	Dihydroquercetin	15.65	11.96	7.60
Negative mode					
1.	5.39	Quinic acid	6.60	3.30	3.16
2.	10.21	β-mangostin	4.58	2.45	4.02
3.	10.82	α-mangostin	4.74	2.04	3.18
4.	14.68	A-type Proanthocyanidin	12.12	7.31	11.01
5.	17.13	Procyanidin B1	21.64	29.43	22.33
6.	19.49	Procyanidin C1	21.57	11.56	20.63
7.	22.04	(-)-Epicatechin	28.49	41.03	26.62
8.	28.08	Procyanidin B1	0.26	2.89	9.05
